# An Integrated Micromirror System for High‐Throughput Multi‐Parametric Cell Imaging

**DOI:** 10.1002/smtd.70813

**Published:** 2026-07-06

**Authors:** Yingchao Meng, Xiaobao Chao, Andrew J. deMello, Stavros Stavrakis

**Affiliations:** ^1^ Department of Chemistry and Applied Biosciences Institute for Chemical and Bioengineering Zürich Switzerland; ^2^ Guangzhou National Laboratory Guangzhou China

**Keywords:** fabrication, fluorescence, microfluidics, nanoscopic scale, photolithography, stereolithography, stroboscope, taxonomy

## Abstract

Imaging flow cytometry combines the high‐throughput capabilities of conventional flow cytometry with single‐cell morphological analysis, enabling the extraction of high‐content information from large and heterogeneous cell populations. Whilst powerful, current imaging flow cytometers rely on complex optical assemblies to achieve high detection sensitivities and spatial resolution. Optofluidic systems that monolithically integrate microfluidics and optical components offer a route to both simplifying and miniaturizing imaging flow cytometers, whilst enhancing imaging performance. Herein, we present a sheathless optofluidic imaging flow cytometry platform (optoIFC) that incorporates microfabricated mirrors for simultaneous two‐color fluorescence and brightfield imaging. The use of two‐photon stereolithography enables the single‐step fabrication of micromirrors directly within microfluidic channels, substantially simplifying device production. The use of such systems with stroboscopic fluorescence illumination provides for imaging throughputs exceeding 3,000 cells per second. To showcase the efficacy of the optoIFC, TNF‐α–induced NF‐κB nuclear translocation in HL‐60 cells is investigated through dual‐colour fluorescence imaging, confirming that the optoIFC enables precise cell sizing and high‐content mapping of intracellular heterogeneity across large populations at high‐throughput.

## Graphical abstract

A sheathless optofluidic platform integrates 3D‐printed micromirrors for simultaneous brightfield and dual‐color fluorescence imaging. Leveraging two‐photon stereolithography for rapid, single‐step fabrication, the system enables high‐content intracellular mapping and translocation analysis at throughputs exceeding 3000 cells per second. This miniaturized approach streamlines high‐resolution morphological profiling and enhances the detection of heterogeneity in large cell populations.

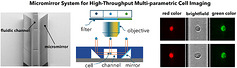

## 1 Introduction

Flow cytometry (FC) is widely recognized as the gold‐standard technique for high‐throughput enumeration of single‐cells in immunology, cancer biology and disease monitoring [1, 2, 3]. Although conventional FC is capable of analyzing single‐cells at rates approaching 10 000 cells/s [4], it does not generate spatially‐resolved information. To address this limitation, imaging flow cytometry (IFC) has emerged as a powerful alternative method for probing heterogeneous cellular populations. The combination of the high‐throughput, multiparametric capabilities of conventional FC with the high‐quality morphological information accessible through optical microscopy means that IFC offers a unique approach to cellular analysis. Indeed, the rapid extraction of high‐content information from single‐cell images enables the quantification of various targets (such as proteins and nucleic acids) across subcellular compartments (such as the nucleus and mitochondria) [4, 5, 6]. The Cytek Amnis ImageStreamX Mk II imaging flow cytometer can successfully capture high‐resolution fluorescence images of single‐cells in flow [7]. Operation relies on the use of a time delay and integration (TDI) high‐speed charge‐coupled device (CCD) camera (rather than the photomultiplier tubes used in contemporary flow cytometers) to provide for high‐resolution imaging with excellent sensitivity [8]. Despite a favorable throughput of approximately 5000 cells per second (at 20x magnification) [6, 7], the Cytek Amnis ImageStreamX Mk II IFC is significantly more expensive than conventional flow cytometers, making it far less accessible to end users. Additionally, because the translation of a cell is synchronized with the vertical charge transfer of each pixel in the camera sensor, TDI read out necessitates precise fluidic control to ensure that cells move at a defined speed without rotation [8]. This makes integration of downstream sorting highly challenging, as even minor flow fluctuations will induce imaging instabilities [3, 6]. Moreover, the Cytek Amnis ImageStreamX Mk II IFC system requires large amounts of sheath fluid to hydrodynamically focus the sample into a narrow stream, both increasing the cost per experiment and restricting its application in settings where sample volume is limited. Finally, analysis is time‑consuming due to the need for extensive system calibration prior to acquisition.

To tackle the aforementioned issues, recent activities have focused on the development of microfluidic‐based imaging flow cytometry platforms. Such technologies offer precise control over fluid flows, which facilitates the efficient analysis of micron‐sized objects in a high‐throughput fashion. For example, Han and Lo introduced a spatial–temporal transformation method that reconstructs cell images from signals acquired by photomultiplier tubes (PMTs), thereby adding imaging capabilities to a microfluidic platform [9]. Whilst an interesting innovation, the approach lacks multi‑parametric analysis capabilities and operates at relatively low throughput. To preserve image quality when cells are moving at high linear velocities, Lei et al. employed optofluidic time‑stretch microscopy, achieving an analytical throughput of over 10 000 cells per second with sub‑micron resolution [10]. Unfortunately, this method relies on complex optical modules, custom electronics, and extensive digital signal processing, resulting in a bulky, costly, and mechanically sophisticated system. To eliminate motion blur caused by the extended exposure times of CCD or CMOS cameras, Mikami et al. developed an optomechanical imaging method integrating a speed‑controlled polygon scanner. This approach delivers virtual‑freezing fluorescence images at a throughput exceeding 10 000 cells per second and at 20× magnification [11], but does require the use of a series of timing control circuits, significantly increasing operational complexity.

Recently, our group developed a microfluidic IFC that utilizes inertial focusing to align cells into a single stream and stroboscopic fluorescence imaging to capture “blur‐free” images of rapidly moving cells [12]. Through parallelization, the system achieved a throughput of 20 000 cells/s at 20× magnification. Subsequently, we reported a second generation ultra‐high‐throughput imaging flow cytometer designed for multiparametric fluorescence imaging [13]. This innovative system utilized a viscoelastic fluid to focus cells [14] and stroboscopic light‑sheet illumination, enabling high‑throughput operation (11 000 cells/s at 20× magnification), whilst maintaining a spatial resolution close to the diffraction limit.

Recent advances in imaging flow cytometry have pushed the boundaries of speed and dimensionality. For instance, Ugawa et al. developed a high‑throughput parallel optofluidic 3D‑imaging flow cytometer based on an oblique‑plane‑microscopy light‑sheet technique, enabling three‐dimensional (3D) imaging [15]; Additionally, the same group showed that stroboscopic light‑sheet excitation combined with optofluidic spatial transformation can capture 3D fluorescence images of cells flowing at over 10 m/s [16]. In addition, Son et al. reported a portable light‑sheet optofluidic microscope for 3D fluorescence imaging flow cytometry, achieving subcellular 3D resolution with a throughput of approximately 1000 cells/s [17]. Although these platforms demonstrate impressive throughput and imaging performance, a common limitation is the need to use complex optical setups for multiparametric imaging. This complexity inherently restricts both practical utility and widespread adoption. In addition, many of these systems necessitate rigorous post‐fabrication alignment between the fluidic chip and detection optics.

In recent years, several optofluidic systems have been reported for single‑cell analysis [18, 19]. For example, Holzner and co‐workers presented an optofluidic system in which microlens arrays were integrated and used for parallel brightfield imaging flow cytometry [20]. Specifically, microlens arrays were fabricated on glass coverslips using UV lithography and thermal reflow. Such structures substantially improved image resolution (compared to lens‑free configurations) without sacrificing the accessible field of view, enabling high‑throughput imaging at rates up to 50 000 cells/s. In a related work, Cao and colleagues developed a lens–mirror optofluidic system for ultra high‑throughput single‑droplet detection [21]. In this setup, a spherical cavity was integrated into the fluidic channel and coated with a silver layer that functions as a mirror. On the opposite side of the channel, a microlens was positioned in line with the opposing mirror. This mirror–lens configuration amplified fluorescence signals from individual droplets by more than two orders of magnitude, enabling high‑speed detection at rates up to 40 000 droplets/s. Additionally, optofluidic platforms for multiparametric detection, based on simultaneous measurement of scattered light and fluorescence, have been developed. For instance, Hengoju and co‐workers designed an optofluidic detection system for microbiological analysis in droplets, incorporating three pairs of microlenses and optical fibers to collect fluorescence, absorbance, and scattering signals [22]. These examples illustrate the advantages of optofluidic devices for high‑throughput micro‑object imaging.

Despite progress, the number of integrated optofluidic systems for IFC remains limited. Koh et al. reported a prism–mirror‑embedded microfluidic device capable of simultaneously capturing top‑ and side‑view images of cells [23], serving as a high‐speed and cost‐effective 3D imaging platform. Unfortunately, reflected side‑view images were 16% larger than top‑view images, a discrepancy caused by light‑path redirection within the device. Miura et al. demonstrated light‑sheet excitation of flowing cells in a mirror‑embedded microfluidic device, using an aluminum‑coated glass mirror to reflect the light sheet into the microchannel [24]. Such an approach was successful in extracting fluorescence images of rapidly moving adenocarcinoma cells at throughputs of up to 10 000 cells/s. More recently, Lee et al. developed a micromirror‑embedded coverslip that allows 3D imaging of cells in both brightfield and fluorescence modes [25]. Here, micromirrors were fabricated using multiple microfabrication steps, including mechanical slicing, soft lithography, and metal deposition. Importantly, all the mirror‑embedded microfluidic systems described above require precise alignment between micromirrors and microfluidic channels, posing significant challenges during device fabrication.

Accordingly, there is a recognized need for integrated optofluidic devices that can be easily and reliably fabricated while at the same time providing high‐resolution images. To this end, we present an optofluidic imaging flow cytometry platform (optoIFC) for multiparametric cell imaging. Two‑photon stereolithography is employed to fabricate both 3D micromirrors and microchannel moulds in a single step, greatly simplifying chip manufacturing and minimizing alignment errors [21, 26, 27]. Stroboscopic fluorescence illumination is then used to enable blur‑free cellular imaging at high linear velocities [12, 13]. The optoIFC simultaneously acquires two‑colour fluorescence (red and green, R‑FL and G‑FL) and brightfield (BF) images of cells flowing at speeds up to 0.054 m/s. To demonstrate utility, we perform image‑based analysis of NF‑κB nuclear translocation in HL‑60 cells, achieving a throughput exceeding 3000 cells/s.

## 2 Results and Discussion

### 2.1 OptoIFC Design and Operation

**FIGURE 1:**
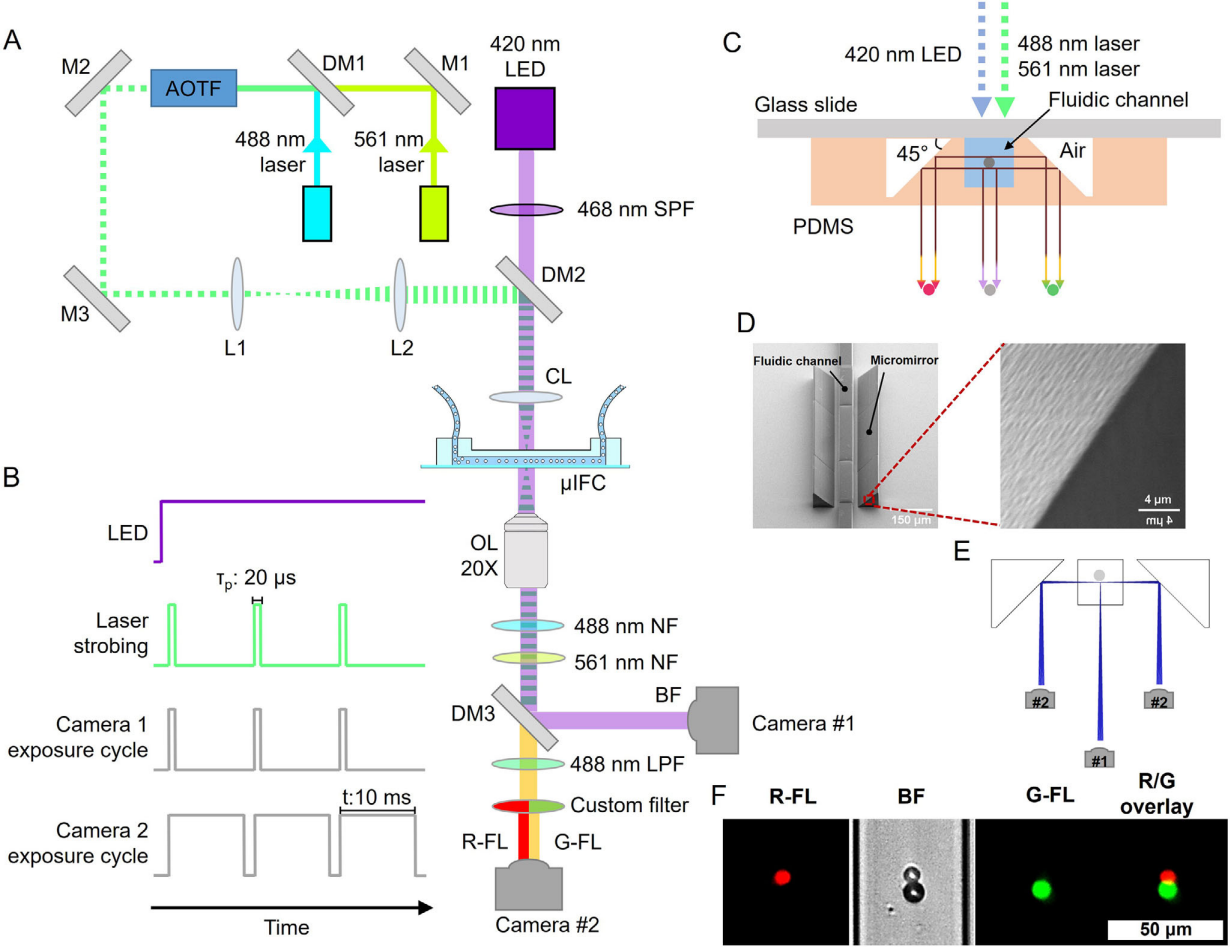
Schematic of the high‐throughput multi‐parametric optoIFC. (A) The optical setup provides for both two‐color fluorescence (R‐FL and G‐FL) and brightfield imaging. Two laser sources (488 and 561 nm) were used for fluorescence excitation, along with a 420 nm LED light source for brightfield imaging. Camera #1 captures brightfield images, while camera #2 collects R‐FL and G‐FL images. (B) The output signals from camera #2 trigger camera #1 and an AOTF. The image acquisition rate of camera #2 was set to 100 frames/s, and laser strobing was set to 20 µs. The exposure time for camera #1 was set to 20 µs. (C) Sideview of the microfluidic device. Micromirrors oriented at an angle of 45° ensure total light reflection of emitted light. (D) SEM images of the 3D printed microfluidic mold used for device fabrication. (E) Simulated ray trajectories calculated using the COMSOL Multiphysics ray‐tracing module highlight varying optical paths for reflected and non‐reflected light. These differences require distance adjustments for each camera (1 and #2) to ensure in‐focus images. (F) A series of images captured by the optoIFC.

Schematics of the optoIFC are presented in Figure 1 and Figure S1. Figure 1A reports the entire optical setup for two‐colour fluorescence and brightfield imaging. 488 and 561 nm excitation beams are combined using a dichroic mirror (DM1) and then passed through an acousto‐optic tunable filter (AOTF), expanded, and collimated using a telescope, and shaped into a light sheet (Figure S2) using a cylindrical lens (CL). The collimated laser excitation beams are directed toward the microfluidic channel via reflection by a polychromatic dichroic mirror (DM2) and subsequently transformed into a light sheet by a cylindrical lens positioned prior to the excitation objective. A purple LED light source (420 nm) is used for brightfield illumination. Photons are collected through a 20× objective lens and spectrally filtered using two notch filters (NF) at 488 nm and 561 nm. Photons reflected by a third dichroic mirror (DM3) are detected by camera #1 for BF imaging, whilst transmitted photons are further filtered by a 488 nm long pass filter (LPF) and a custom red/green bandpass filter before being imaged onto camera #2. The transmission profile of the custom red/green bandpass filter is shown in Figure S3. To obtain blur‐free fluorescence images, the optoIFC incorporates stroboscopic illumination [12, 13]. Here, synchronization between excitation pulses and the camera integration period is achieved by using the output of camera #2 to trigger both camera #1 and the acousto‐optic tunable filter (Figure 1B). The image acquisition rate of camera #2 was set to 100 frames/s. Limited by the camera sensor areas and the magnification of the objective, the horizontal field of view (detection area) was set to 540 µm (Figure S1). This length is chosen so that the transit time of a cell across the detection region matches the camera frame period. At this velocity, each cell is captured in a single frame, avoiding both under‐sampling (where a rapidly moving cell passes through the detection region without being imaged) and over‐sampling (where a cell appears in multiple consecutive frames). Matching the flow velocity to the camera frame rate enables robust, blur‑free image acquisition at high throughput.

Since the fluidic channel has a cross section of 50 µm × 50 µm, the optimum flow rate is approximately 8 µL/min. The exposure time for camera #1 was set to 20 µs and the laser strobe time (pulse length) was also set to 20 µs. This yields a theoretical motion blur of approximately 1 µm, a value comparable to the lateral resolution of the 20× 0.50 NA objective lens (0.7 µm at 600 nm). Because the light sheet concentrates the available laser power into a small volume (the focal plane), the excitation intensity is much higher than in wide‐field mode. This allows the use of extremely short exposure times (20 µs pulses) to freeze the motion of rapidly moving cells (5.4 cm/s) whilst still generating sufficient fluorescence for the CMOS sensor to detect. Figure 1C,D shows the structure of the microfluidic device in the imaging zone. Given that the real part of the refractive index of the substrate (PDMS) is 1.42 [28], the micromirrors were aligned 45° to the channel bottom, ensuring total internal reflection at the PDMS/air interface (Figure 1C, Text S1). Scanning electron microscope (SEM) images provide an excellent view of the fabricated microchannel and mirrors, highlighting a small degree of surface roughening (Figure 1D), arising from the layer‑by‑layer 3D printing process. The impact of surface roughness on image quality is a critical consideration for any integrated optical system. In this work, the two photon lithography parameters were optimized (scanning speed and laser power) to ensure a surface roughness below the wavelength of visible light. The staircase effect, a common artifact in additive manufacturing, was suppressed by utilizing sub‐micron layer heights (200–500 nm), resulting in highly specular surfaces. While residual nano‐scale roughness may contribute to a slight increase in background “haze” due to diffuse reflection, our experimental results demonstrate that the spatial resolution remains sufficient to resolve sub‐cellular events, such as the translocation of NF‐κB into the nucleus (see “TNF‐α‐induced NF‐κB translocation in HL‐60 cells” section). This roughness could be further minimized by varying the photoresist formulation and printing parameters, such as slicing distance, hatching distance, laser power, printing speed, and development time [29, 30, 31]. Figure 1E shows simulated ray trajectories at the center of the fluidic channel, calculated using the *Ray Optics Module* of COMSOL Multiphysics. Since light reflected by the micromirrors travels a longer distance in path #2 (used for two‐color fluorescence imaging) than in path #1 (used for brightfield imaging), the two cameras are positioned at slightly different distances from the optofluidic device to accommodate the distinct image focusing paths. Figure 1F shows representative two‐color fluorescence and brightfield images of polystyrene beads. The image on the far‐left shows a red fluorescent (R‑FL) bead with a diameter of 7.32 µm (as specified by the vendor). The second image shows a brightfield image of two beads of different sizes. The third image shows a green fluorescent (G‑FL) bead with a diameter of 9.77 µm, with the final image being an overlay of the R‑FL and G‑FL channels.

### 2.2 OptoIFC Characterization

To evaluate the sensitivity of the optoIFC, suspensions of five fluorescent bead populations (containing the same fluorophore but with different surface densities) were analyzed. Single‑file focusing of beads was achieved using a viscoelastic 0.3% w/v 600 kDa polyethylene oxide carrier fluid. The vendor‑specified fluorescence intensities of the five bead populations are 0, 14 642, 28 570, 289 055, and 587 060 MESF (molecules of equivalent soluble fluorophore, FITC) units. Analysis of the calibration bead populations was used to generate a fluorescence calibration curve shown in Figure S4. For an average volumetric flow rate of 8 µL min^−1^ and a 488 nm laser power of 800 mW, the limit of detection (LOD) was determined to be 7722 MESF units. For most flow cytometry applications, fluorescence intensities typically exceed 30,000 MESF units, confirming that the optoIFC provides excellent sensitivity for routine cellular assays [13, 32].

**FIGURE 2:**
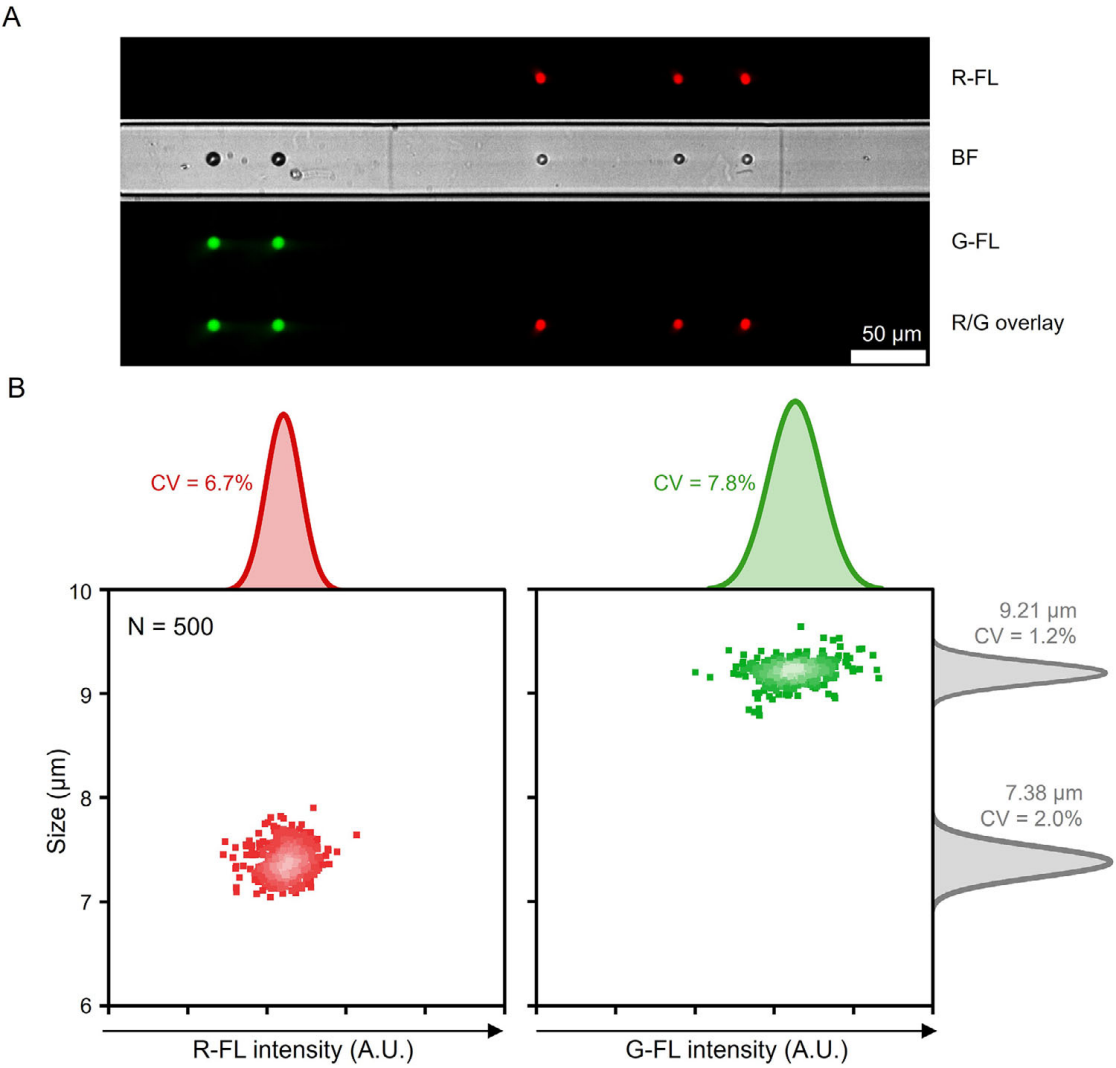
Microparticle analysis using the optoIFC (A) Representative single‐frame images acquired by the optoIFC, showing red‐fluorescence (R‐FL), brightfield (BF), green‐fluorescence (G‐FL), and an overlay of R‐FL and G‐FL channels for 7.32 µm R‐FL and 9.77 µm G‐FL polystyrene beads. (B) Scatter plots of particle size versus fluorescence intensity and associated size distributions of both R‐FL and G‐FL particles. The average diameters of R‐FL and G‐FL beads are calculated to be 7.38 µm (CV = 2.0%) and 9.21 µm (CV = 1.2%), respectively. The CV values, obtained from the fluorescence intensity distributions, are 6.7% for the R‐FL PS beads and 7.8% for the G‐FL PS beads.

To further evaluate the performance of the optoIFC, we analyzed a mixed population of commercially available polystyrene beads (9.77 µm diameter green‐fluorescent beads and 7.32 µm diameter red‐fluorescent beads) as mammalian cell mimics. Figure 2 presents two‐color fluorescence and brightfield images of the particles and the associated scatter plots of particle size versus fluorescence intensity. Representative R‑FL, brightfield, G‑FL, R‑FL/G‑FL images are provided in Figure 2A. Assay precision and reproducibility were assessed through calculation of the coefficient of variation (CV) of both particle size and fluorescence intensity distributions (Figure 2B). The CV is defined as the ratio of the standard deviation of the Gaussian distribution to the mean for a given population. Particle size distributions were extracted directly from brightfield images, while fluorescence intensity distributions were derived from the R‑FL and G‑FL channels. Measured average diameters were 7.38 ± 0.15 µm for the R‐FL PS beads and 9.21 ± 0.11 µm for the G‐FL PS beads; values that are in excellent agreement with the vendor‑specified values of 7.32 µm and 9.77 µm, respectively.

To further validate the quantitative reliability of the optoIFC platform, we performed benchmark experiments using a conventional (non‐imaging) flow cytometer (Figure S5). Specifically, CV values, derived from the particle size distributions, of 2.0% for R‐FL PS beads and 1.2% for G‐FL PS beads, are comparable to those obtained with conventional flow cytometry (2.1% and 2.2%), as shown in Figure S5. CV values derived from the fluorescence intensity distributions were 6.7% for the R‐FL PS beads and 7.8% for the G‐FL PS beads; values in good agreement with those extracted from conventional flow cytometry (Figure S5). It should be noted that the CV values for the fluorescence intensity distributions are larger than those for the particle size distributions. This is due to the fact that internal dye loading is directly proportional to the particle volume, leading to an increased variability in intensity measurements. Overall, these results demonstrate that the optoIFC is able to accurately quantify both particle size and fluorescence intensity distributions from images, with performance comparable to that of conventional flow cytometry.

### 2.3 TNF‐α‐Induced NF‐κB Translocation in HL‐60 Cells

Subsequently, the optoIFC was used to monitor the translocation of the nuclear factor kappa B (NF‐κB) in human leukemic HL‐60 cells activated by the tumor necrosis factor alpha (TNF‐α). NF‐κB plays a crucial role in regulating various aspects of innate and adaptive immune functions and acts as a central mediator of inflammatory responses [33]. In mammalian cells, the NF‐κB family consists of five members, including NF‐κB1 (p50), NF‐κB2 (p52), RelA (p65), RelB and c‐Rel [34], of which the NF‐κB p50/p65 heterodimer is considered the most abundant and widely studied NF‑κB complex [35]. To detect and quantify NF‑κB translocation from the cytoplasm to the nucleus, we employed the Amnis NF‑κB Translocation Kit. Here, the nucleus is stained with 7‑AAD (red fluorescence), while NF‑κB p50 is labeled with an anti‑human NF‑κB antibody conjugated to Alexa Fluor 488 (green fluorescence).

**FIGURE 3:**
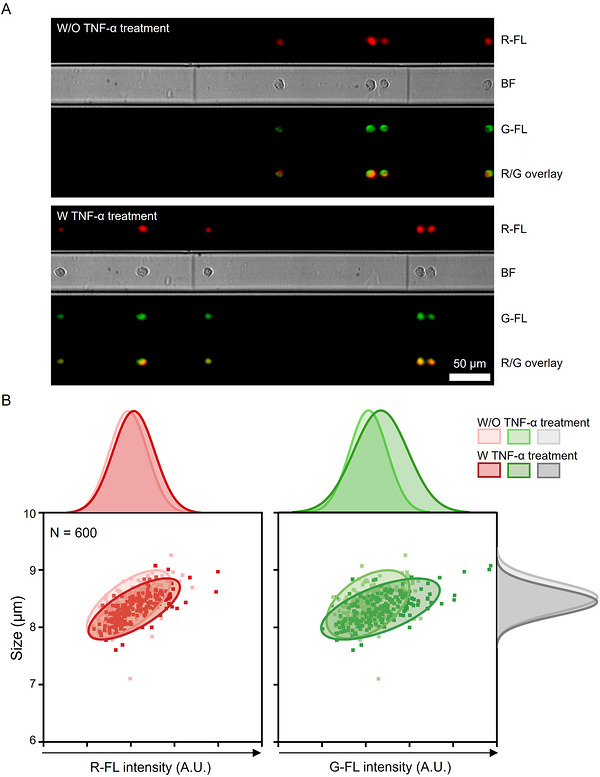
TNF‐α‐induced NF‐κB translocation analysis using the optoIFC. (A) Representative single‐frame images of HL‐60 cells acquired without (top) and with (bottom) TNF‐α treatment. In the absence of TNF‐α, NF‐κB p50 (G‐FL) is predominantly cytoplasmic; upon TNF‐α stimulation, G‐FL signal translocates into the nucleus, as evidenced by strong colocalization with the nuclear stain (R‐FL). (B) Scatter plots of cell size versus fluorescence intensity, together with size and fluorescence intensity distributions for R‐FL and G‐FL signals under both conditions. After TNF‐α treatment, only a minor shift is observed in the cell size and G‐FL intensity distributions, confirming that the increased colocalization reflects nuclear translocation rather than changes in overall cell size or total fluorescence intensity.

TNF‐α is a pleiotropic cytokine with a wide range of biological effects including inflammation, host defense, upregulation of adhesion molecules, proliferation, differentiation and apoptosis [36]. In this assay, TNF‑α treatment induces nuclear translocation of NF‑κB, which is monitored using the optoIFC. Experiments were performed at a flow rate of 8 µL/min, and 800 mW power for both 488 nm and 561 nm. For the translocation assay, HL‐60 cells were stimulated with 10 ng/mL TNF‐a for 30 min, following standard manufacturer protocols. We analyzed 100% of the cells captured within the field of view, utilizing only a minimal automated filtration step to exclude non‐cellular debris. Figure 3 presents a series of cell images and the corresponding cell size and fluorescence intensity distributions with (W) and without (W/O) TNF‐α treatment. Specifically, Figure 3A displays representative images of untreated (top) and TNF‐α‐treated (bottom) HL‐60 cells. Upon TNF‐α stimulation, a significant increase in the overlap between green fluorescence (G‐FL, NF‐κB p50) and red fluorescence (R‐FL, nucleus) is observed, consistent with NF‐κB translocation from the cytoplasm to the nucleus. In contrast, only a minor shift is visible in the cell size versus fluorescence intensity distributions between the two conditions (Figure 3B). These findings were further validated using conventional flow cytometry, which showed no significant differences in cell size and fluorescence intensity distributions for both conditions (Figure S6). This confirms that the observed changes in signal overlap are due to altered subcellular localization (translocation) rather than changes in the overall cell size or total fluorescence intensity.

**FIGURE 4:**
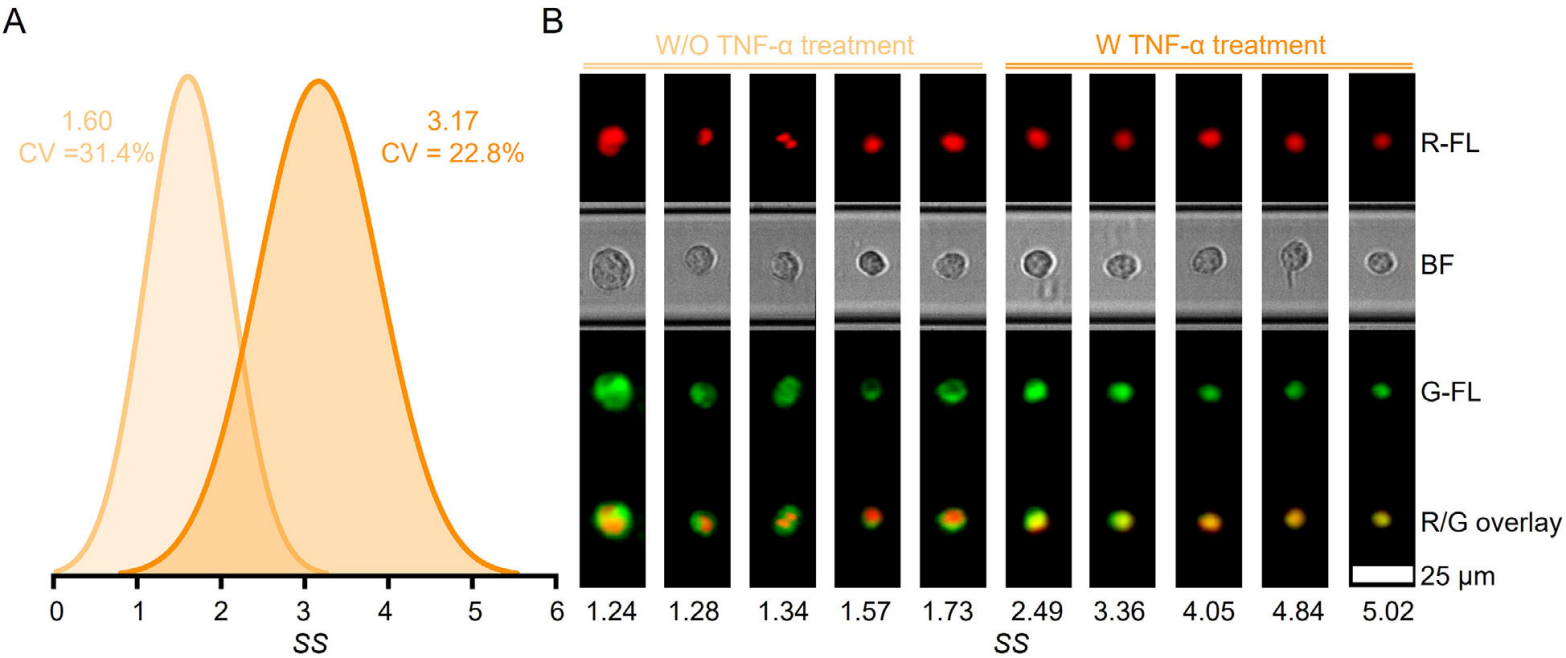
Similarity score (SS) distributions and representative cell images from the optoIFC analysis of NF‐κB translocation. (A) SS distributions for HL‐60 cells with and without TNF‐α treatment. Upon TNF‐α stimulation, the average SS increases significantly from 1.60 (untreated) to 3.17 (treated), reflecting enhanced spatial overlap between NF‐κB p50 (G‐FL) and the nucleus (R‐FL), and is consistent with NF‐κB nuclear translocation. (B) Representative images of ten individual cells, acquired under the same conditions and annotated with their corresponding SS values. Increasing SS values correspond to a progressive redistribution of the NF‐κB p50 signal from the cytoplasm into the nucleus, visually confirming the quantitative shift in SS and demonstrating the cellular response to TNF‐α treatment.

To gain a deeper insight into the impact of TNF‐α on NF‐κB nuclear translocation, we defined a similarity score (SS) that quantifies the spatial overlap between the 7‐AAD (R‐FL, nuclear) and NF‐κB p50 (G‐FL) images. SS distributions were employed, as described in the Materials and Methods. SS distributions for cells with and without TNF‐α treatment are shown in Figure 4A, revealing a clear shift upon stimulation: the mean SS increases from 1.60 in untreated cells to 3.17 in TNF‐α–treated cells, indicating enhanced colocalization of NF‐κB with the nucleus. A low SS (close to the untreated population, ∼1–2) reflects minimal overlap between the G‐FL and R‐FL images and thus NF‐κB is largely excluded from the nucleus. In contrast, a high SS (approaching the TNF‑α–treated values, ∼3 or higher) reflects substantial overlap, consistent with NF‑κB nuclear translocation. Figure 4B illustrates ten representative cell images with their corresponding SS values, showing that an increase in SS is accompanied by a progressive redistribution of the G‑FL signal from the cytoplasm into the R‑FL–stained nucleus, visually confirming the quantitative shift in the SS distribution.

Finally, to validate the performance of the optoIFC, we compared NF‐κB translocation results with those obtained using the commercial ImageStreamX system. Figure S7 reports the corresponding similarity score distributions, with average SS values of −0.02 (without TNF‐α) and 3.16 (with TNF‐α) on the commercial platform. The difference in absolute SS values between the optoIFC and the ImageStreamX system is attributed to the use of different image processing algorithms. In this regard, it should be noted that the commercial ImageStreamX system does not disclose details of its image analysis pipeline, making quantitative comparison of SS values impossible. However, while the absolute SS values for the untreated populations differ between the optoIFC and the commercial system (due to variations in pixel‐masking and background‐normalization algorithms), the ability to statistically resolve translocation events is highly consistent across both platforms, as illustrated by the representative cell images shown in Figure 4 and Figure S7. Additionally, Figure S8 presents images of HL‐60 cells acquired with the optoIFC at a throughput of 3000 cells/s, which is comparable to the throughput of the ImageStreamX system at 20× magnification [6, 7].

## 3 Conclusions

Taken together, these results demonstrate that the optoIFC can deliver high‐quality two‐color fluorescence and brightfield (BF) images of single‐cells at high throughput, enabling robust co‐localization analysis on large cell populations. This is achieved through the integration of microfabricated mirrors within a microfluidic channel, which enables simultaneous two‑color fluorescence and brightfield imaging in a compact, sheathless format, eliminating the need for external hydrodynamic focusing and drastically reducing sample consumption. In this regard, the use of two‐photon stereolithography enables rapid, alignment‐free prototyping of both the fluidic channel and optical elements in a single step, significantly simplifying device manufacturing.

The “added value” of the optoIFC lies in the combination of high throughput, multimodal detection, high spatial resolution imaging, and low system cost. The optoIFC platform operates at an analytical throughput exceeding 3000 cells/s, matching the high‐speed performance of the gold‐standard ImageStreamX system at 20× magnification, whilst simultaneously providing an appreciable cost reduction. Alternative high‐throughput strategies typically rely on pixel‐readout optimization, such as reducing the number of active readout pixels or restricting the field‐of‐view. Such approaches do not mitigate the architectural complexity of conventional IFCs, which require external, bulky optical assemblies and rigorous alignment of multi‐channel detection paths. In contrast, the optoIFC leverages monolithic micromirror integration to allow for simultaneous acquisition of both brightfield and two‐channel fluorescence images. By consolidating optical routing in the microfluidic mold, the need for complex and alignment‐sensitive beam‐shaping is eliminated, providing a highly scalable and cost‐effective solution for high‐content, multiparametric single‐cell analysis. The compact, alignment‐free architecture ensures that the spatial correlation between subcellular features imaged in two different colors (e.g. nuclear and cytoplasmic markers) is robustly preserved during high‐speed flow.

In contrast to conventional flow cytometry, which provides only integrated intensity signals, the optoIFC platform delivers high‐content, spatially resolved data and is capable of resolving subtle changes in intracellular composition, as exemplified in the analysis of NF‐κB translocation. Spatial resolution is quantified by the SS that measures the pixel‑wise correlation between the NF‑κB (G‑FL) and nuclear (R‑FL) images, enabling objective, single‑cell assessment of nuclear translocation. When compared to the gold‐standard ImageStreamX imaging flow cytometer, the optoIFC platform provides for enhanced imaging quality at equivalent throughput and achieves comparable analytical performance at a significantly reduced complexity and cost (Table S1). Interestingly, it should be noted that the throughput of the optoIFC could be easily increased through the implementation of passive cell manipulation strategies that ensure uniform cell spacing at high speeds [37, 38, 39] or by adoption of a higher frame‑rate camera.

A key feature of the optoIFC, when compared to other optofluidic systems, is that it can be fabricated through a single stereolithographic process. This enables straightforward, rapid manufacturing of both fluidic and optical components through mold replication and simplifies device production. This also ensures intrinsic alignment between optical and fluidic paths, enabling simultaneous acquisition of brightfield and fluorescence images in a compact, alignment‐free format, and bypassing the rigorous manual calibration required in conventional instruments. The flexibility of two‐photon stereolithography also opens up the possibility of multi‐angular imaging, which could be used for 3D single‐cell imaging in future embodiments. Whilst the current implementation of the optoIFC is a 2D multiparametric system, its “alignment‐free” nature offers a scalable and cost‐effective alternative for high‐content analysis. Beyond additional wavelengths, the scalability of the two‐mirror configuration for two‐color imaging also allows for multi‐angular imaging. Since two‐photon lithography allows for the fabrication of arbitrary 3D geometries, additional micromirrors can be integrated at varying heights, tilt angles, or longitudinal positions along the microchannel. This allows the CMOS camera to be spatially partitioned to capture not only multiple spectral bands but also simultaneous side‐ and top‐views of the same cell. Such a configuration paves the way for 3D tomographic reconstruction in flow, a capability typically requiring complex, multi‐camera setups in discrete optical systems. It is important to note that the integrated micromirrors are inherently broadband. Therefore, the “on‐chip” architecture is not limited to specific wavelengths. Scalability to higher‐order multiplexing would primarily require external adjustments, such as replacing the current dual‐band filter with a multi‐notch filter in the detection path.

Given the rich spatial information obtainable from single‐cell images, the optoIFC platform is well suited for integration with machine‐learning methods with a view to automated, real‐time cell classification in clinical applications [4, 6, 40, 41]. Such a platform would be especially valuable for identifying circulating tumor cells in the context of liquid biopsies [42, 43]. In this regard, we anticipate that the optoIFC will make significant contributions to large‑scale, multiparametric cell analysis, leading to a deeper understanding of cellular behavior in disease processes.

## 4 Materials and Methods

### 4.1 Device Design and Fabrication

Micromirrors were designed and optimized using the *Ray Tracing Module* within COMSOL Multiphysics 5.5 (COMSOL, Burlington, USA). The complete optofluidic device (including both micromirrors and fluidic channels) was designed and modelled in SolidWorks (Dassault Systèmes, Waltham, USA), with the design subsequently being exported and converted into a stereolithographic file format. The entire master structure was fabricated via two‐photon lithography using a Photonic Professional GT femtosecond laser lithography system (Nanoscribe, Karlsruhe, Germany). Initially, an aliquot of IP‐S photoresist (Nanoscribe, Karlsruhe, Germany) was dispensed onto a 25 mm × 25 mm ITO‐coated glass slide. During printing, selected regions of photoresist were exposed to a 780 nm femtosecond laser in a layer‐by‐layer fashion. For structures in the detection region, slicing and hatching distances were set to 0.1 µm, to ensure optimum printing resolution. For the remaining portions of the structure, slicing and hatching distances were set to 1 and 0.5 µm, respectively. A 25× objective lens (Zeiss, Oberkochen, Germany) with a numerical aperture (NA) of 0.8 was used for printing due to its optimal combination of resolution and working distance. After printing, uncured photoresist was removed by development in 1‐methoxy‐2‐propanol acetate (Sigma–Aldrich, Buchs, Switzerland) for 10 min followed by a 2‐min isopropanol (Sigma–Aldrich, Buchs, Switzerland) rinse. The master mold was then baked at 150°C for 6 h.

Subsequently, a 10:1 (wt/wt) mixture of polydimethylsiloxane (PDMS) monomer to curing agent (Sylgard 184, Dow Corning, Midland, USA) was degassed in a desiccator for 60 min, poured over the master mold and cured at 70°C for 4 h [44].The cured PDMS substrate was then carefully peeled off from the master mold. Inlet and outlet ports were created in the structured PDMS substrate using a 22‐gauge puncher (I and Peter Gonano, Niederösterreich, Austria). Finally, the microfluidic device and a 24 × 75 × 1 mm glass slide (Menzel‐Glaser, Braunschweig, Germany) were plasma treated in a Zepto air plasma chamber (Diener Electronic, Ebhausen, Germany) for 1 min and then brought into contact. The entire assembly was then placed on a hot plate at 120°C for 2 h to strengthen bonding of the PDMS substrate to the glass slide. The surface morphology of the master mold was examined using scanning electron microscopy (ULTRA 55, Zeiss, Oberkochen, Germany). Prior to imaging, a thin layer of gold was sputtered onto samples to improve conductivity. The conductive coating improves signal‑to‑noise ratio and resolution by stabilizing electron emission and reducing artifacts, enabling accurate visualization of surface morphology.

### 4.2 Device Operation and Data Acquisition

The microfluidic device was placed on a motorized *xy* translation stage (Mad City Laboratories, Madison, USA) mounted on an inverted Ti‐E microscope (Nikon, Zurich, Switzerland) equipped with two CMOS cameras: camera #1 (iDS UI‐3060CP‐M‐GL Rev. 2, IDS Imaging Development Systems, Obersulm, Germany) for brightfield imaging and camera #2 (ORCA‐flash 4.0, Hamamatsu, Solothurn, Switzerland) for fluorescence imaging. The outputs of a green (561 nm, Coherent Genesis MX, Glasgow, UK) and a blue (488 nm, Coherent Genesis MX, Glasgow, UK) laser were combined using a dichroic mirror DM1 (AHF, Tübingen, Germany). Subsequently, the combined beam was passed through an nC‐400‐650‐TN acousto‐optic tunable filter (AA Opto‐electronic, Orsay, France) and shaped into a line using a LJ1558RM‐A cylindrical lens (Thorlabs, Lübeck, Germany) after being reflected by a polychromatic dichroic mirror DM2 (ZT488/561rpc, AHF, Tübingen, Germany). A 20× objective (Plan Apo 20×, NA 0.50, Nikon, Zurich, Switzerland) was used to collect photons, and a set of filters, including a 488 nm notch filter (F40‐487, AHF, Tübingen, Germany), a 561 nm notch filter (NF561‐18, Thorlabs, Lübeck, Germany), a 488 nm long‐pass (LP) filter (AHF, Tübingen, Germany) and a custom red/green filter (Shenzhen Infrared Laser Technology, Shenzhen, China) was used to spectrally filter the emitted light into red and green fluorescence. A purple LED light source (M420L3, Thorlabs, Lübeck, Germany) was used for brightfield imaging. The purple LED light was initially filtered through a 468 nm short‐pass filter (AHF, Tübingen, Germany), directed by DM2, and ultimately collected on camera #1 after being reflected by DM3 (AT505DC, AHF, Tübingen, Germany). The output signal of camera #2 was used to trigger camera #1 and the AOTF (Figure 1B). The image acquisition rate of camera #1 was set to 100 frames/s. The detection area was defined as a rectangular region of 540 µm × 50 µm within the microchannel (Figure S1). The laser strobe time was set to 20 µs. The exposure time for camera #1 was also set to 20 µs. To ensure each cell or bead was imaged only once per frame, the average volumetric flow rate was maintained at 8 µL/min.

Polystyrene bead or cell suspensions were loaded into a 1 mL syringe (Hamilton Laboratory Products, Reno, USA) and delivered into the device at the desired flow rate using a neMESYS precision syringe pump (Cetoni, Korbussen, Germany). In all experiments, beads and cells were suspended in a viscoelastic PEO carrier solution. A commercial calibration kit (Quantum Alexa Fluor 488 MESF, Bangs Laboratories, Fishers, USA) comprising five microsphere populations labelled with increasing amounts of the same fluorophore, was used for calibration measurements. For microparticle experiments, 9.77 µm diameter green fluorescent (G‐FL) PS beads (FC06, Bangs Laboratories, Fishers, USA) and 7.32 µm diameter red fluorescent (R‐FL) PS beads (FSSY007, Bangs Laboratories, Fishers, USA) were used. HL‐60 cells were stained using the Amnis NF‐κB translocation kit (Luminex, Austin, USA), following the manufacturer's instructions.

### 4.3 Viscoelastic Fluid Preparation and Characterization

Viscoelastic stock solutions were prepared by fully dissolving 600 kDa PEO (Sigma–Aldrich, Buchs, Switzerland) in 1×PBS buffer (Thermo Fisher Scientific, Reinach, Switzerland) to a concentration of 1% (w/v). Stock solutions were aged at room temperature for 1 week to ensure a uniform viscosity [45]. Prior to each experiment, a 0.3% (w/v) PEO concentration was prepared by diluting the 1% (w/v) PEO stocksolution with 1×PBS.

### 4.4 Flow Cytometry Measurements

Conventional flow cytometry measurements were performed on a CytoFLEX flow cytometer (Beckman Coulter, Pasadena, USA). Forward scatter (FSC‐H) signal vs fluorescence signal emitted at 585 nm (PE‐H) was to gate rreen fluorescence (R‐FL) signals. FSC‐H vs fluorescence signal emitted at 525 nm (FITC‐H) was used to gate G‐FL signals. For all measurements, the sample flow rate was set to 30 µL/min. Data were acquired and processed using CytExpert software (Beckman Coulter, Pasadena, USA). Additional data processing was performed using FlowJo software (FlowJo, Ashland, USA).

Control IFC measurements were conducted using a Cytek Amnis ImageStreamX Mk II imaging flow cytometer (Millipore, Schaffhausen, Switzerland). When using the ImageStreamX imaging flow cytometer, each “Ch” label refers to a fixed spectral detection band. Ch01, Ch05 and Ch02 were chosen for BF, R‐FL, and G‐FL imaging, respectively. For all measurements, the sample flow rate was set to “moderate”. Results were analyzed using IDEAS 6.2 software (Millipore, Schaffhausen, Switzerland).

### 4.5 Image Analysis

Post‐processing of data was performed using algorithms that extract cell size and intensity from fluorescence and brightfield images. Image processing algorithms were implemented within MATLAB (Mathworks, Natick, USA). Figure S9 shows the image analysis steps for BF, R‐FL, and G‐FL images. Specifically, the background was first subtracted from the original image, and image contrast adjusted to improve visibility. A Gaussian filter was then applied to reduce noise and smooth irregularities [46]. The processed image was then converted into a binary image and any gaps in the binary image filled to create solid, connected regions. Erosion and dilation operations were performed to fine‐tune cell boundaries. Finally, objects that were either too small or too large (based on a priori information) were removed from the processed image. Cell size information was then extracted from the BF image, and R‐FL and G‐FL intensities were calculated based on the R‐FL and G‐FL images, respectively. Data obtained from the processing algorithms were then plotted as scatterplots and histograms using OriginPro 2021b (OriginLab, Northampton, USA).

### 4.6 Preparation of HL‐60 Cells

HL‐60 cells were cultured in RPMI 1640 medium containing L‐Glutamine (Life Technologies, Paisley, UK), supplemented with 10% (v/v) fetal bovine serum (Thermo Fisher Scientific, Zurich, Switzerland), and 50 U/mL penicillin (Sigma–Aldrich, Buchs, Switzerland). All cell cultures were sustained in a 5% CO_2_ humidified atmosphere at 37°C. Prior to experimentation, cells were washed with1×PBS and counted using conventional flow cytometry.

### 4.7 Calculation of Similarity Score (*SS)*

The *SS* is defined as:

(1) $$SS=ln\left(\frac{1+\rho }{1-\rho }\right)$$

where ρ is the Pearson correlation coefficient between the two image intensity matrices. For a 2D image matrix, ρ is calculated according to,

(2) $$\rho =\frac{\sum_{m}\sum_{n}\left(A_{mn}-\bar{A}\right)\left(B_{mn}-\bar{B}\right)}{\sqrt{\left[\sum_{m}\sum_{n}{\left(A_{mn}-\bar{A}\right)}^{2}\right]\left[\sum_{m}\sum_{n}{\left(B_{mn}-\bar{B}\right)}^{2}\right]}}\;$$

Here *A* is the 2D matrix of image *A*, $\bar{A}$ is the average value of *A*, *B* is the 2D matrix of image *B*, $\bar{B}$ is the average value of *B*, *m* is the number of pixels in width, and *n* is the number of pixels in length.

## Conflicts of Interest

The authors declare no conflicts of interest.

## Supporting information




**Supporting File**: smtd70813‐sup‐0001‐SuppMat.pdf.

## Data Availability

The data that support the findings of this study are available on request from the corresponding author. The data are not publicly available due to privacy or ethical restrictions.
