# Supplementary material for: An Integrated Micromirror System for High‐Throughput Multi‐Parametric Cell Imaging
Source: Small Methods. 2026 Jul 6;10(15):e70813. doi: 10.1002/smtd.70813 (PMC13450170; doi:10.1002/smtd.70813)
Supplement: Supplementary file 1 [file SMTD-10-e70813-s001.pdf]

An Integrated Micromirror System for High-Throughput Multi-Parametric Cell Imaging

Yingchao Meng[1,#], Xiaobao Chao[1,2,#], Andrew J. deMello[1,*] and Stavros Stavrakis[1,*]

1. Institute for Chemical and Bioengineering, Department of Chemistry and Applied Biosciences, ETH Zürich, 8093 Zürich, Switzerland.

2. Guangzhou National Laboratory, Guangzhou, China

[#] these authors contributed equally to this paper.

[*]**corresponding author**: andrew.demello@chem.ethz.ch, stavros.stavrakis@chem.ethz.ch

Supplementary Text 1: Total internal reflection (TIR) Analysis

To validate the efficiency of the 45° PDMS micromirrors, we analysed the Total Internal Reflection (TIR) conditions. For the Sylgard 184 PDMS formulation used in this study (Sylgard 184, 10:1 base:curing agent ratio) the refractive index, *n,* is 1.42 at the visible wavelengths used (500-600 nm). This yields a critical angle, θ_c, of 44.8°at the PDMS/air interface. Accordingly, a nominal 45° mirror angle provides a narrow geometric margin of 0.23°. Although this margin is narrow, the system's robustness is significantly enhanced by the refractive index transition from the aqueous medium ($n = 1.33$) into the PDMS ($n = 1.42$), which causes an angular compression effect at the water/PDMS interface. According to Snell's Law, rays entering the higher-index PDMS are refracted toward the normal, compressing the angular cone of emitted light before it reaches the mirror surface. This angular compression ensures that the vast majority of optical flux arrives at the micromirror at angles exceeding the TIR threshold ($\theta_i > \theta_c$), effectively compensating for the narrow nominal margin. *COMSOL Ray Optics* simulations (**Figure 1E**) confirm that under these conditions high reflectance is maintained across the full angular range of collected light.

The impact of fabrication-induced surface roughness on optical performance was evaluated using the Rayleigh criterion for specular reflection. A surface is considered optically smooth when its peak-to-valley roughness (*h*) satisfies the relationship:

$$h < \lambda/8cos(\theta_i)$$

For a peak emission of 550 nm and an incidence angle of 45°, the threshold for specular reflection is approximately 97 nm. SEM characterization of the fabricated facets indicates a peak-to-valley roughness in the sub-100 nm range, confirming that the mirrors operate in the specular regime with negligible diffuse scattering. Furthermore, the large scale of the mirror structures (tens of microns) renders the total integrated fluorescence collection relatively insensitive to minor manufacturing tolerances in the facet angle, as further supported by the COMSOL simulations

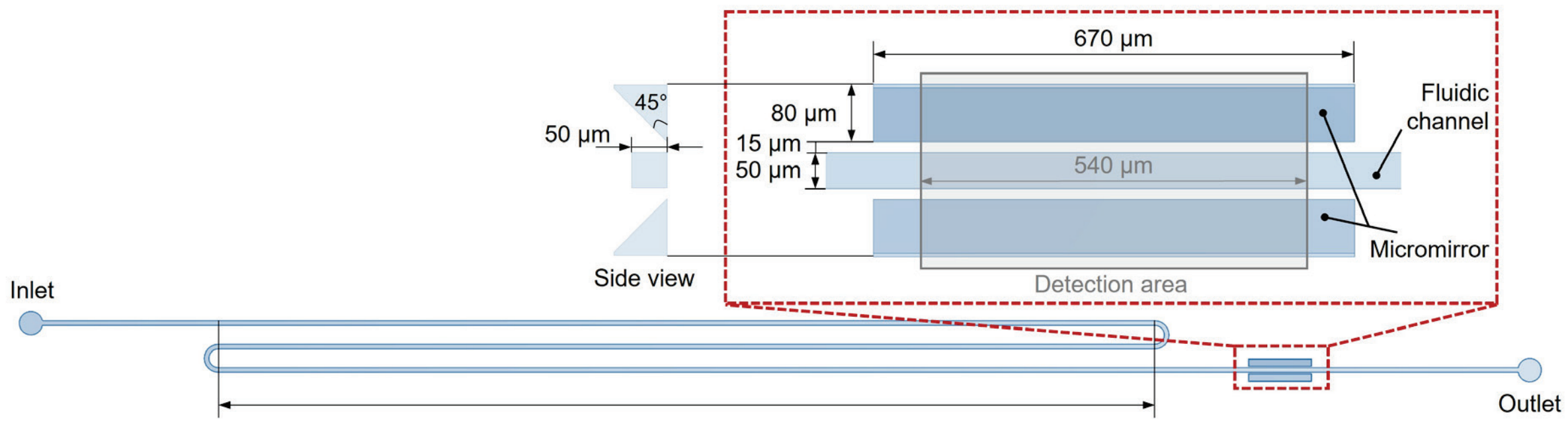

Figure S1. Schematic of the optoIFC device. The fluidic channel has a cross section of 50 µm × 50 µm (width × height). The total length of the fluidic channel is 30 mm. Micromirrors in the detection zone are 670 µm long and 80 µm wide and oriented at an angle of 45° to the plane.

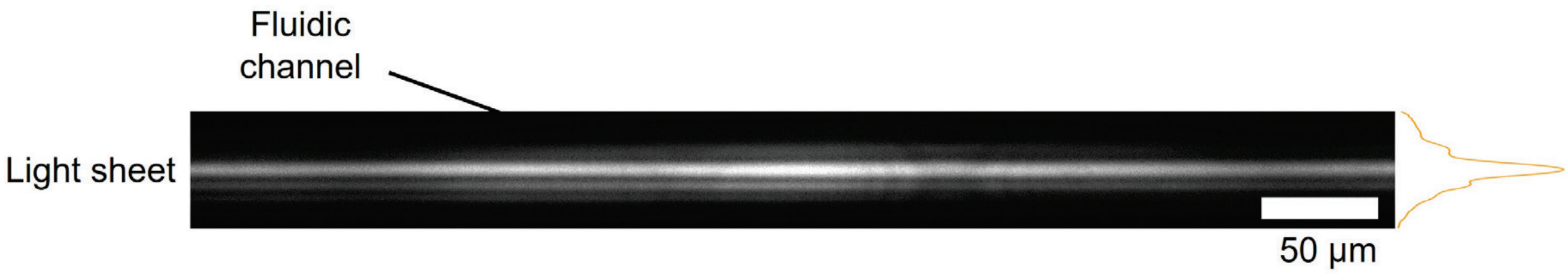

Figure S2. Image of the light sheet captured by the fluorescence camera. The fluidic channel was filled with a FITC solution to allow visualization of the light sheet profile. When the excitation optics generate the light sheet, only the volume (within the channel) intersected by that sheet is excited and able to fluoresce.

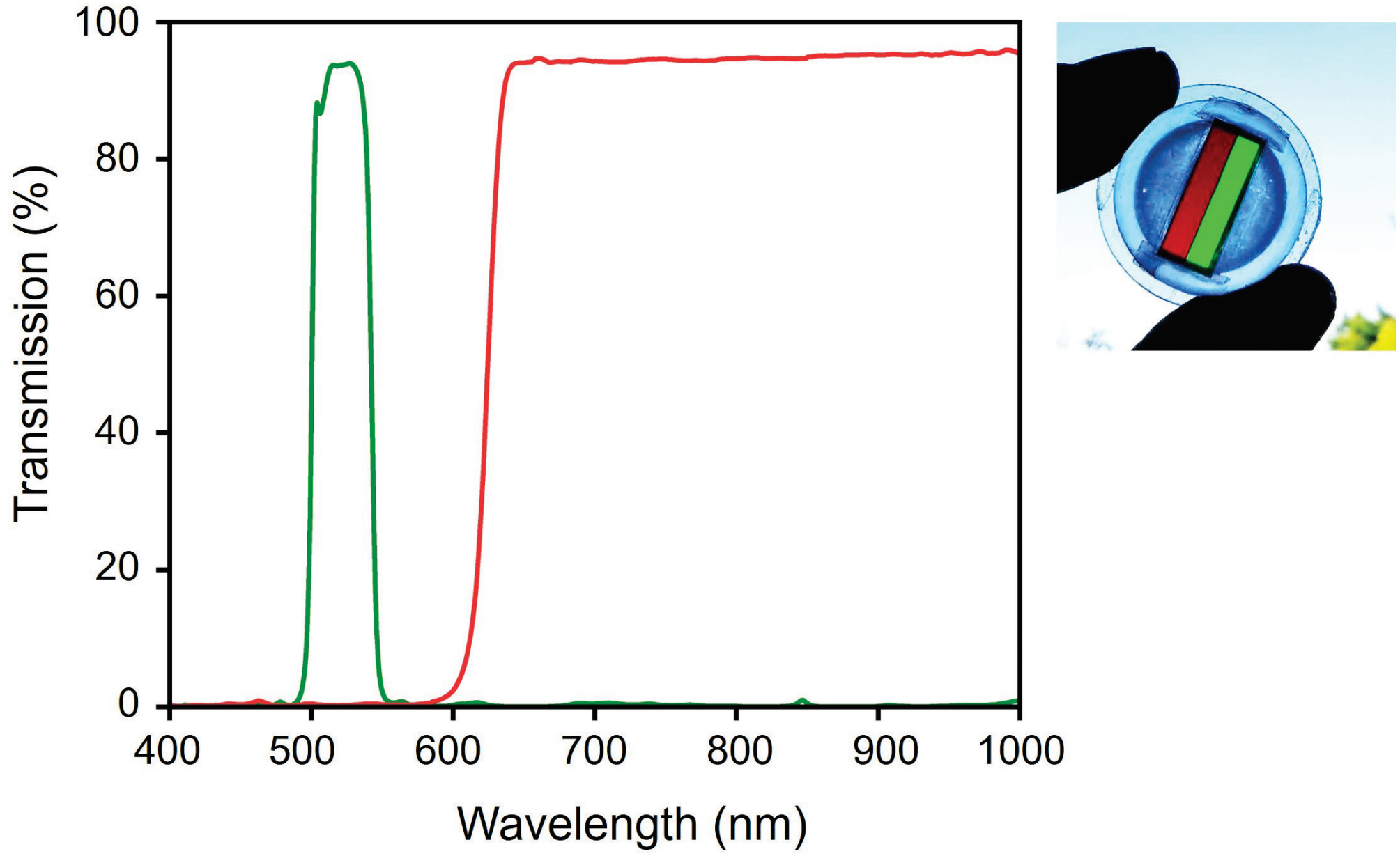

Figure S3. Transmission spectrum of the customized dual-band emission filter. The green-fluorescence (G-FL) channel is defined by a 520 nm bandpass feature, and the red-fluorescence (R-FL) channel by a 620 nm long-pass feature.

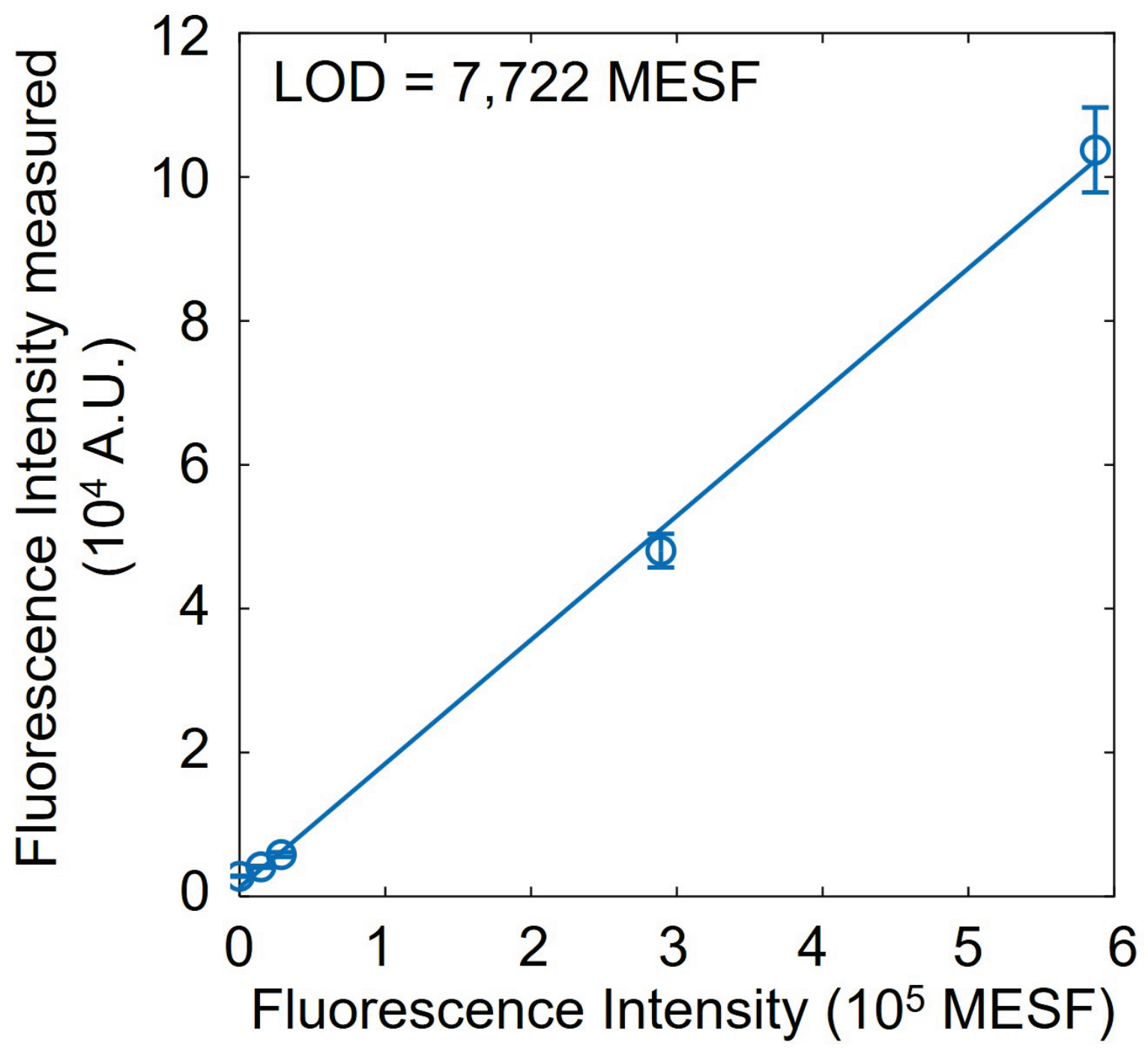

Figure S4. Evaluation of the optoIFC sensitivity. Five different bead populations containing increasing amounts of fluorophore (0, 14,642, 28,570, 289,055, and 587,060 MESF units) were used to construct the calibration curve. The LOD for the optoIFC was determined to be 7,722 MESF. The LOD was defined as the lowest MESF level at which the bead population could be statistically distinguished from the blank (0 MESF) based on their fluorescence intensity distributions. More specifically LOD was calculated using the relationship: $LOD = I_{blank} + 3\sigma_{blank}$ where I_{blank} is the mean intensity of the background (areas of the channel without cells) and σ_{blank} is the standard deviation of that background. Data points represent the mean fluorescence intensity of each bead population and error bars indicate one standard deviation.

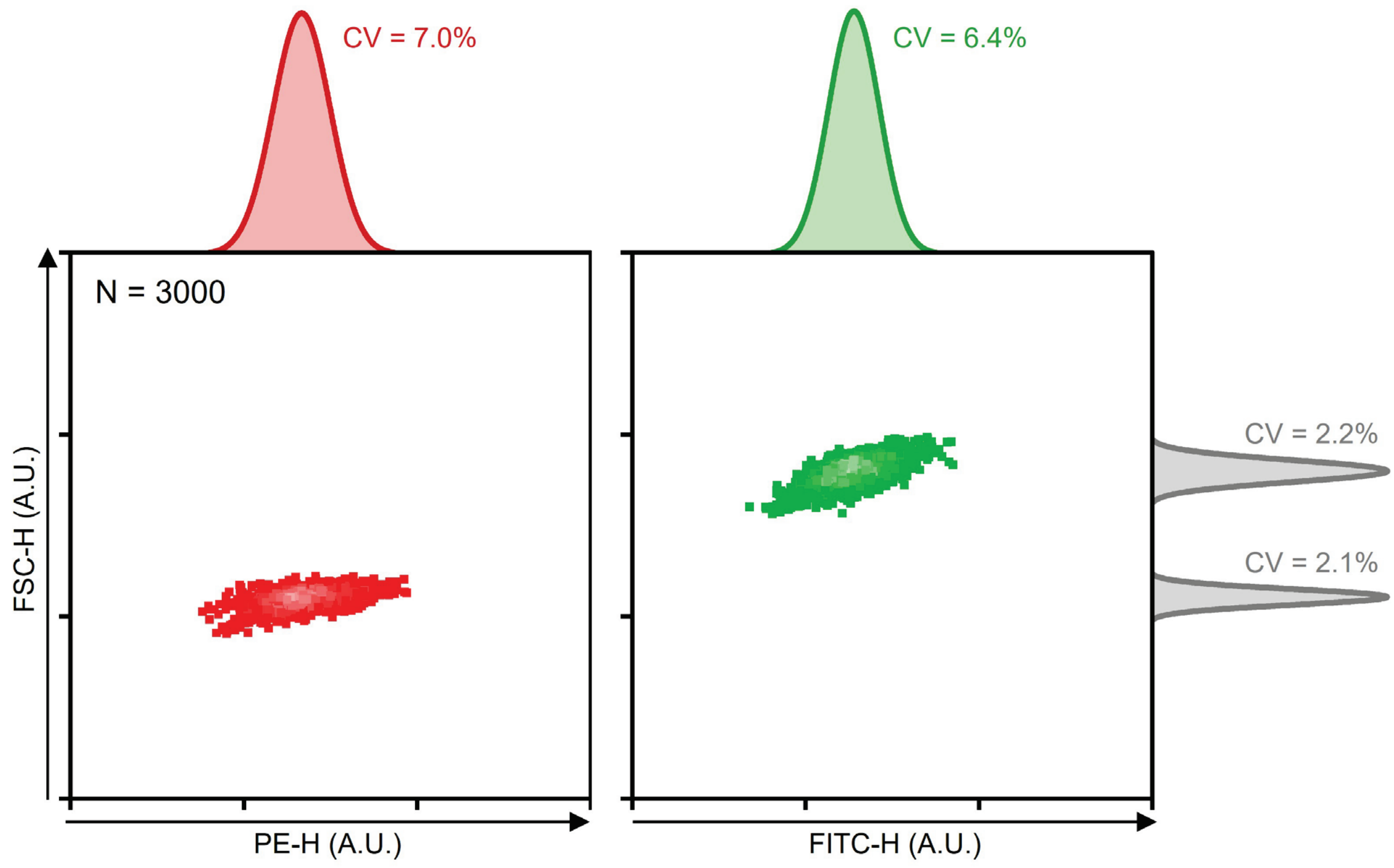

Figure S5. Particle analysis using conventional flow cytometry. FSC-H vs PE-H was used to gate R-FL signals. FSC-H vs FITC-H was used to gate G-FL signals. CV values derived from the bead size histograms were 2.1% and 2.2% for R-FL and G-FL PS beads, respectively. The CV values derived from the fluorescence intensity distributions PE-H and FITC-H were 7.0% for R-FL and 6.4% for G-FL PS beads, respectively.

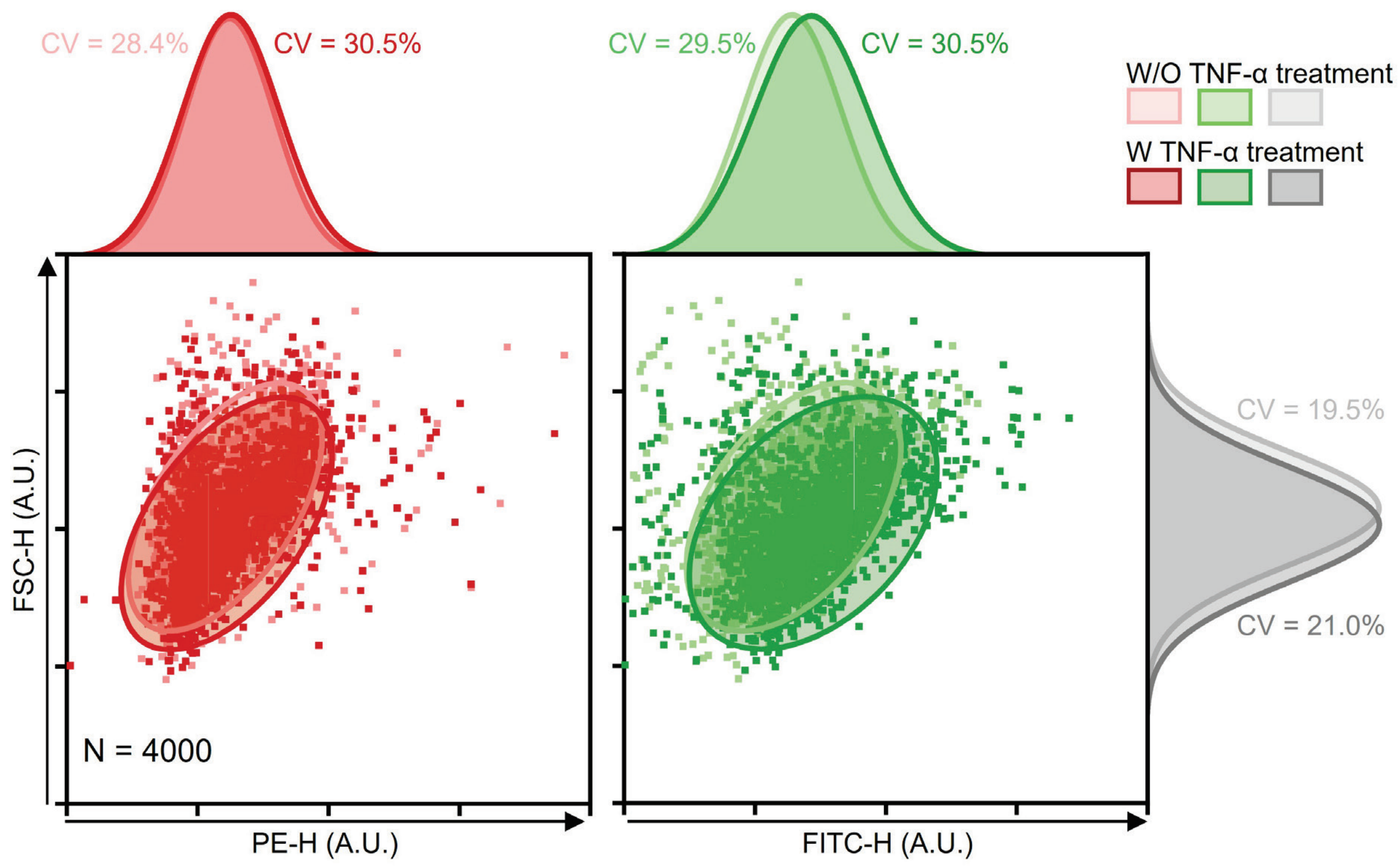

Figure S6. TNF-α-induced NF-κB translocation analysis using a conventional flow cytometer. After TNF-α treatment, only minor shifts are observed in the cell size and fluorescence intensity distributions.

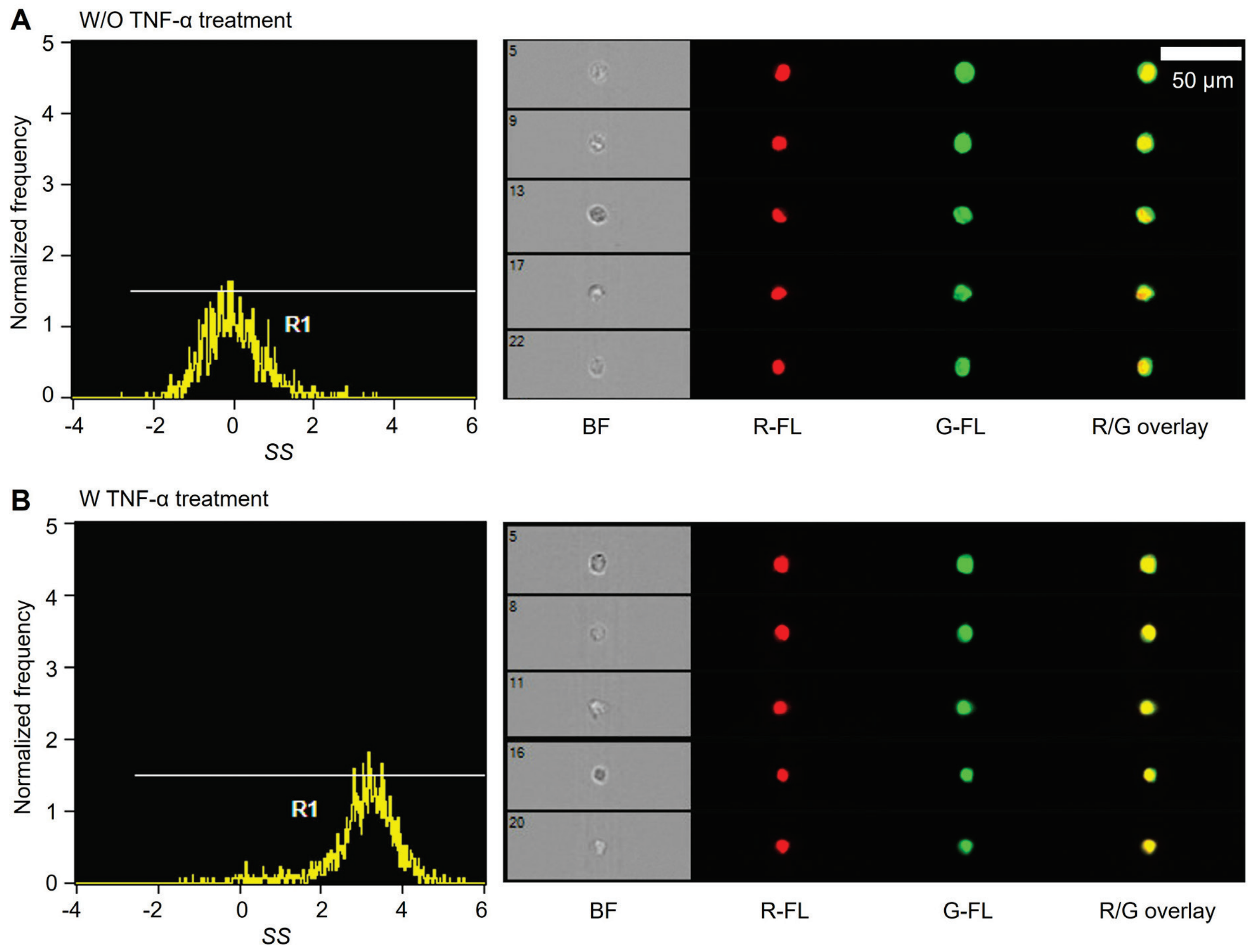

Figure S7. *SS* values obtained using the Cytek® Amnis® ImageStream®X Mk II imaging flow cytometer. After TNF-α treatment, the average *SS* increases from (A) -0.02 to (B) 3.16. From left to the right, images depict BF, G-FL, R-FL and the overlay of R/G of cells.

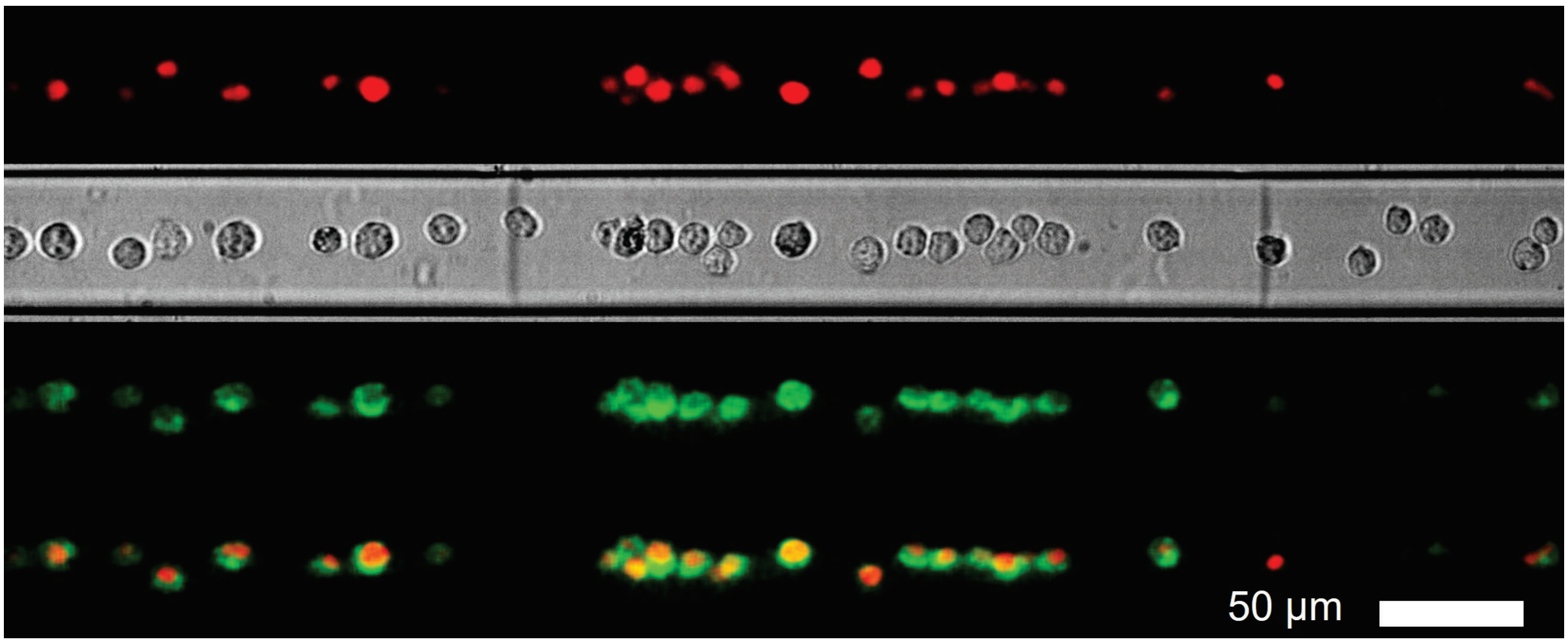

Figure S8. A single image frame demonstrating the capability of the optoIFC to efficiently process a dense concentration of HL-60 cells, at rates exceeding 3000 cells/s.

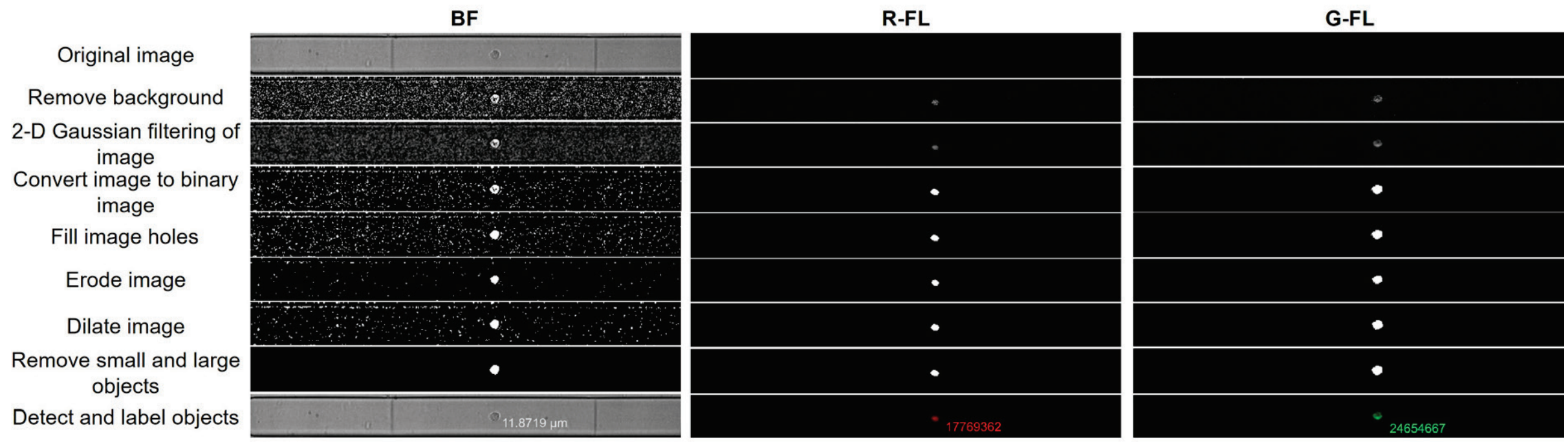

Figure S9. Step-by-step processing of BF, R-FL, and G-FL images. The raw image was first subjected to background subtraction and contrast adjustment. Following this, Gaussian filtration, binary conversion, gap filling, erosion, dilation, and removal of small and large object algorithms were used to isolate objects of interest.

Parameter	optoIFC (This work)	ImageStream®X II (20×)
**Lateral Resolution**	~1.2 µm	~1.0 µm
**Numerical Aperture (NA)**	0.45 (Air)	0.50 (Air)
**Throughput (Analytical)**	>3,000 cells/s	~2,000 - 3,000 cells/s
**NF-κB SS Shift (Δ)**	1.57 (1.60 to 3.17)	3.18 (-0.02 to 3.16)
**Optical Configuration**	monolithic / integrated	discrete / bulk optics
**Alignment Requirement**	none (pre-aligned)	daily calibration required

Table S1. Comparison of optical and analytical specifications between optoIFC and the commercial ImageStream®X II system.
